# Mitochondrial Targeting Domain Homologs Induce Necrotic Cell Death Via Mitochondrial and Endoplasmic Reticulum Disruption

**DOI:** 10.4014/jmb.2104.04021

**Published:** 2021-05-20

**Authors:** Junghee Park, Ji-Hye Han, Seung-Hyun Myung, Hea-jong Chung, Jae-il Park, Ju-Yeon Cho, Tae-Hyoung Kim

**Affiliations:** 1 Department of Biochemistry and Molecular Biology, Chosun University School of Medicine, Gwangju 61452, Republic of Korea; 2 Gwangju Center, Korea Basic Science Institute, Gwangju 61168, Republic of Korea; 3 Department of Medicine, Chosun University Hospital, Gwangju 61453, Republic of Korea

**Keywords:** MTD, MTD homologs, necrotic cell death, mitochondria

## Abstract

The mitochondrial targeting domain (MTD) of Noxa contributes to its mitochondrial localization and to apoptosis induction. As a peptide, MTD fused with octa-arginine (R8), a CPP, induces necrosis related to intracellular calcium influx and destruction of mitochondria and endoplasmic reticulum. We searched for homologs of MTD, and compared their cell killing capability when fused with R8. Three of the seven peptides triggered cell death with similar mechanisms. The comparative analysis of peptide sequences showed that four amino acid sites of MTD are critical in regulating necrosis, suggesting the potential to generate artificial, adjustable cytotoxic peptides, which could be effective medicines for many diseases. Thus, homologs functionality could hint to the functions of their belonging proteins.

## Introduction

Noxa protein belongs to the Bcl-2 family and consists only of a Bcl-2 homology 3 (BH3) domain as well as a mitochondrial targeting domain (MTD) [1, 2]. In response to intolerable micro-environmental perturbations, such as DNA damage and hypoxic conditions, Noxa is transcriptionally induced [3]. The MTD delivers the BH3 domain to the mitochondria to interact with other members of the antiapoptotic Bcl-2 family, including Mcl1 and Bcl2a1, and activates mitochondrial outer membrane permeabilization [2-4]. This induces the cytosolic release of proteins, cytochrome-c and Smac, which activates the caspase system to precipitate apoptosis [5].

The MTD, in addition to its targeting function, strongly induces necrotic cell death with its flanking region (extended MTD, eMTD) or cell-penetrating peptide, octa-arginine (R8) [6, 7]. Within few minutes after the treatment with the MTD peptide, intracellular calcium concentration increases, and extracellular calcium penetrates through the mitochondrial permeability transition (mPT) pore. With the formation of blebs, the calcium concentration decreases and mitochondrial reactive oxidative species (ROS) begin to increase. MTDs also induce mitochondrial swelling, fragmentation, and endoplasmic reticulum (ER) disruption. These processes are completely blocked by 4,4-diisothiocyanatostilbene-2,2′-disulfonate (DIDS), which inhibits the penetration of MTD in the cell and its interaction with VDAC2 [6-8]. The mutations scanned in MTD peptides imply the importance of leucine residues [9, 10].

Bnip1 MTD-like motif (B1MLM), derived from another BH3-only protein, Bnip1, also induces cell death in the same manner [11]. Bnip1 interacts with proapoptotic Bcl-2 family proteins and the adenoviral antiapoptotic protein, E1B 19 kDa [12, 13]. The BH3 domain of Bnip1 is closely involved in apoptosis and mitochondrial fission carried out by Drp1 [14]. B1MLM has a similar arrangement of leucine residues as that of MTD. Although Bnip1 is originally known as a proapoptotic protein, when fused with R8, B1MLM induces rapid necrotic cell death. Similar to MTD, B1MLM induces a sharp rise in calcium concentration and mitochondrial ROS. Additionally, this calcium influx is derived from the extracellular space through mPT pores [11].

In this study, we searched for more MTD homologs and analyzed their sequences and cell death activity. Some of them showed strong cytotoxicity accompanied by intracellular calcium influx, formation of membrane blebs, generation of mitochondrial ROS, and ER destruction. Analysis of their sequences evidenced several critical amino acid sites, and suggested that artificially produced cytotoxic MTD peptides could control cytotoxicity. Moreover, it showed the possibility that proteins containing these homologs may be involved in cell death processes.

## Materials and Methods

### Cell Culture

HeLa cells were purchased from the Korean Cell Line Bank (Korea) and cultured in Dulbecco’s modified Eagle’s medium (DMEM) containing 10% fetal bovine serum and 1× penicillin-streptomycin (100 IU/ml and 100 μg/ml, Gibco-Thermo Fisher, USA) at 37°C in a humidified 5% CO_2_ atmosphere.

### Peptide Synthesis

All the peptides in Table 1 were synthesized by solid phase peptide synthesis and purified by high-performance liquid chromatography to over 95% purity (Anygen, Korea). The peptides were then dissolved in distilled water and stored at -20°C.

### Sequence Alignment

All the sequences of homologs were aligned using Clustal Omega and ClustalW base/residue numbering. The sequences were sorted according to their percent identity with MTD. Each amino acid was colored according to the ClustalX color scheme.

### MTS Assay

HeLa cells were cultured in 96-well plates to 90% confluency and treated with peptides mixed in phenol red-free DMEM for 1 h. After incubation, MTS solution (20 μl, Promega, USA) was added and the absorbance of each well was measured at 450 nm.

### Confocal Microscopy

HeLa cells were cultured on a Lab-Tek Chamber glass slide to 60% confluency. Fluo-4 AM (5 μM, Thermo Fisher) and MitoSOX (5 μM, Thermo Fisher), used to monitor intracellular calcium and mitochondrial ROS, respectively, were mixed in HBSS buffer (0.49 mM MgCl_2_, 0.41 mM MgSO_4_, 5.33 mM KCl, 0.44 mM KH_2_PO_4_, 4.17 mM NaHCO_3_, 137.93 mM NaCl, 0.34 mM Na_2_HPO_4_, 5.56 mM D-glucose, and 1.26 mM CaCl_2_; Gibco-Thermo Fisher) and treated for 10 min. The cells were washed with pre-warmed HBSS buffer and treated the peptides. Immediately after the treatment, we obtained time-lapse images using an argon laser scanning confocal microscope (Leica TCS SP5 Microsystems of KBSI Gwangju Center) at 5-s intervals, 488 nm excitation, to visualize Fluo-4-AM and MitoSOX.

## Results and Discussion

### MTD Homologs Were Searched by Protein Basic Local Alignment Search Tool (BLAST)

**Fig. 1:**
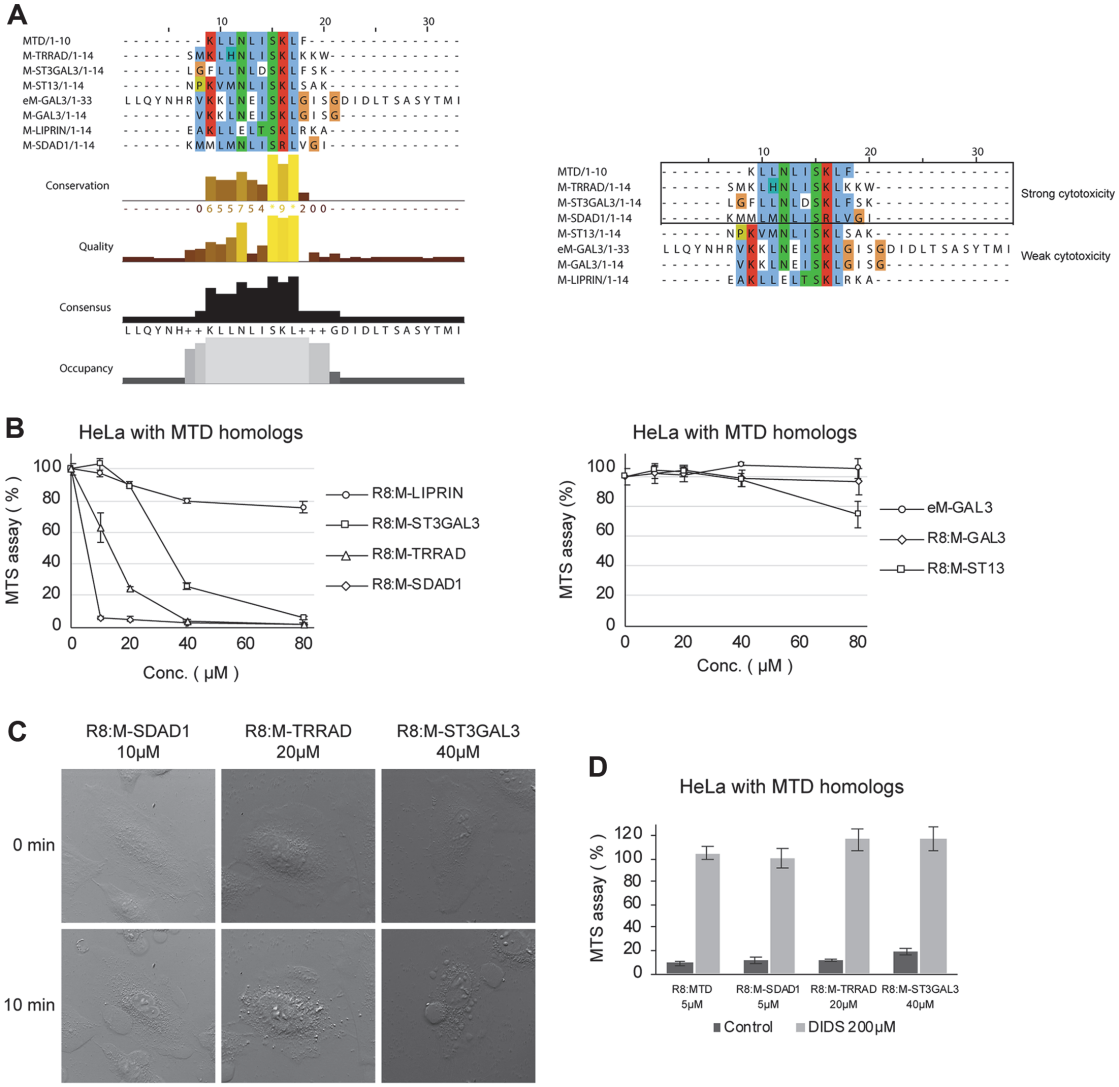
Mitochondrial targeting domain (MTD) homologs induce necrotic cell death as in MTD. **A**. (Left panel) There are several mitochondrial targeting domain (MTD) homologs in human genes. They were searched by protein basic local alignment search tool (BLAST) on National Center for Biotechnology Information (NCBI) and sorted according to percent identity with MTD. Each amino acid was colored according to the ClustalX color scheme (hydrophobic, blue; positive charge, red; negative charge, magenta; polar, green; glycine, orange; proline, yellow; aromatic, cyan; cysteine, pink). (Right panel) The cytotoxicity of mitochondrial targeting domain (MTD) homologs is determined by its amino acid sequence. MTD homologs were grouped according to their cytotoxicity, then sorted according to percent identity with MTD. Each amino acid was colored according to the ClustalX color scheme (hydrophobic, blue; positive charge, red; negative charge, magenta; polar, green; glycine, orange; proline, yellow; aromatic, cyan; cysteine, pink). **B**. Cell viabilities were determined by MTS assay 1 h after treating HeLa cells with MTD homologs fused with octa-arginine (R8). HeLa cells treated with R8:M-LIPRIN, R8:MST3GAL3, R8:M-TRRAD, and R8:M-SDAD1(left panel). HeLa cells treated with eM-GAL3, R8:MGAL3, and R8:M-ST13 (right panel). **C**, HeLa cells treated with R8:M-SDAD1 (5 μM), R8:M-TRRAD (20 μM), and R8:M-ST3GAL3 (40 μM), then observed for 10 min using differential interference contrast microscopy. **D**, HeLa cells were treated with R8:M-SDAD1 (10 μM), R8:M-TRRAD (20 μM), and R8:M-ST3GAL3 (40 μM), with and without DIDS (200 μM); after 1 h, cell viability was determined by MTS assay.

In a previous study, MTD and B1MLM showed comparable cell killing activity and process [11]. We assumed that more MTD-like domains exist and searched for them using protein BLAST on National Center for Biotechnology Information (NCBI). As a result, several proteins were found to contain an MTD-like domain, which, for convenience, were marked as “M-Proteins”, for example, M-SDAD1. These MTD homologs were aligned and sorted according to their percent identity with MTD (Fig. 1A). We selected the MTD homologs with at least 60% sequence identity to 10 MTD amino acids sequences (Table 1). In particular, we found that eM-GAL3 has a flanking region similar to that of eMTD.

### MTD Homologs Induce Necrotic Cell Death as in MTD

MTD and B1MLM peptides induce necrotic cell death when associated with R8. R8 is a well-known cell penetrating motif [7, 11]. In previous study, we have shown that MTD peptide without R8 did not show any cell killing activity, which indicates that MTD peptide itself cannot penetrate the barrier of cell membrane [9]. To evaluate the cytotoxicity of MTD homologs, we synthesized these peptides and fused R8 at the N-terminus. As the concentration increased, the cytotoxicity of R8:M-TRRAD, R8:M-ST3GAL3, and R8:M-SDAD1 were dramatically increased, whereas the treatment with R8:M-ST13, R8:M-GAL3, eM-GAL3, and R8:M-LIPRIN hardly altered cell viability (Fig. 1B). R8:M-SDAD1 was the most cytotoxic, followed by R8:M-TRRAD and R8:M-ST3GAL3. Cytotoxic peptides induced membrane blebs and precipitated necrotic cell death in 10 min (Fig. 1C). In the previous studies, we have shown that the leucine residues at the 5^th^ and 9^th^ sites of MTD play critical roles in the necrosis-inducing activity of MTD. The replacement of leucine residues at these sites with alanine significantly reduced the necrosis-inducing activity of R8:MTD [9, 10]. The glutamate residues at 6^th^ and 13^th^ sites of M-GAL3 and eM-GAL3, respectively, which correspond to the leucine residues at 5^th^ of MTD, may explain the low cytotoxicity of these peptides (Fig. 1A). In addition, the phenylalanine at 10^th^ of MTD played an important role in the necrosis-inducing activity of MTD as well [9, 10]. The residues at 12^th^ of M-ST13 and M-LIPRIN, which correspond to the hydrophobic phenylalanine residue at 10^th^ of MTD, is replaced with the polar amino acid serine residue of M-ST13 and the charged arginine residue of M-LIPRIN, and these changes may cause the low cytotoxicity of these peptides. Together, we believe that changes in amino acids at the corresponding critical sites like 5^th^, 9^th^, or 10^th^ of MTD in M-ST13, eM-GAL3, M-GAL3, and M-LIPRIN may explain the low cytotoxic activity of these peptides.

The cytotoxicity of the peptides was completely inhibited by DIDS as R8:MTD, which implies that they induce cell death with the same mechanism (Fig. 1D). The difference in intensities between cytotoxic peptides also provided further clues (Fig. 1A, right panel). According to the comparison of amino acid composition and cytotoxicity, the hydrophobicity of isoleucine (sixth) and phenylalanine (tenth) could influence cytotoxicity. Interestingly, M-SDAD1 showed the lowest identity with MTD, but also the strongest cytotoxicity. The close match of the side chains of M-SDAD1 and MTD shows that their polarity is important in determining the cytotoxicity of the MTD homologs, suggesting that cytotoxicity could be controlled by replacing specific amino acids.

### Cytotoxic MTD Homologs Induce Intracellular Calcium Influx

Calcium is an important regulator of necrosis induced by MTD [6, 7]. R8 induces intracellular calcium spikes with cargos, while MTD and B1MLM increase intracellular calcium influx [15]. Although the exact mechanism is not certain, chelation of intracellular calcium by BAPTA-AM inhibited MTD-induced cell death [9].

**Fig. 2:**
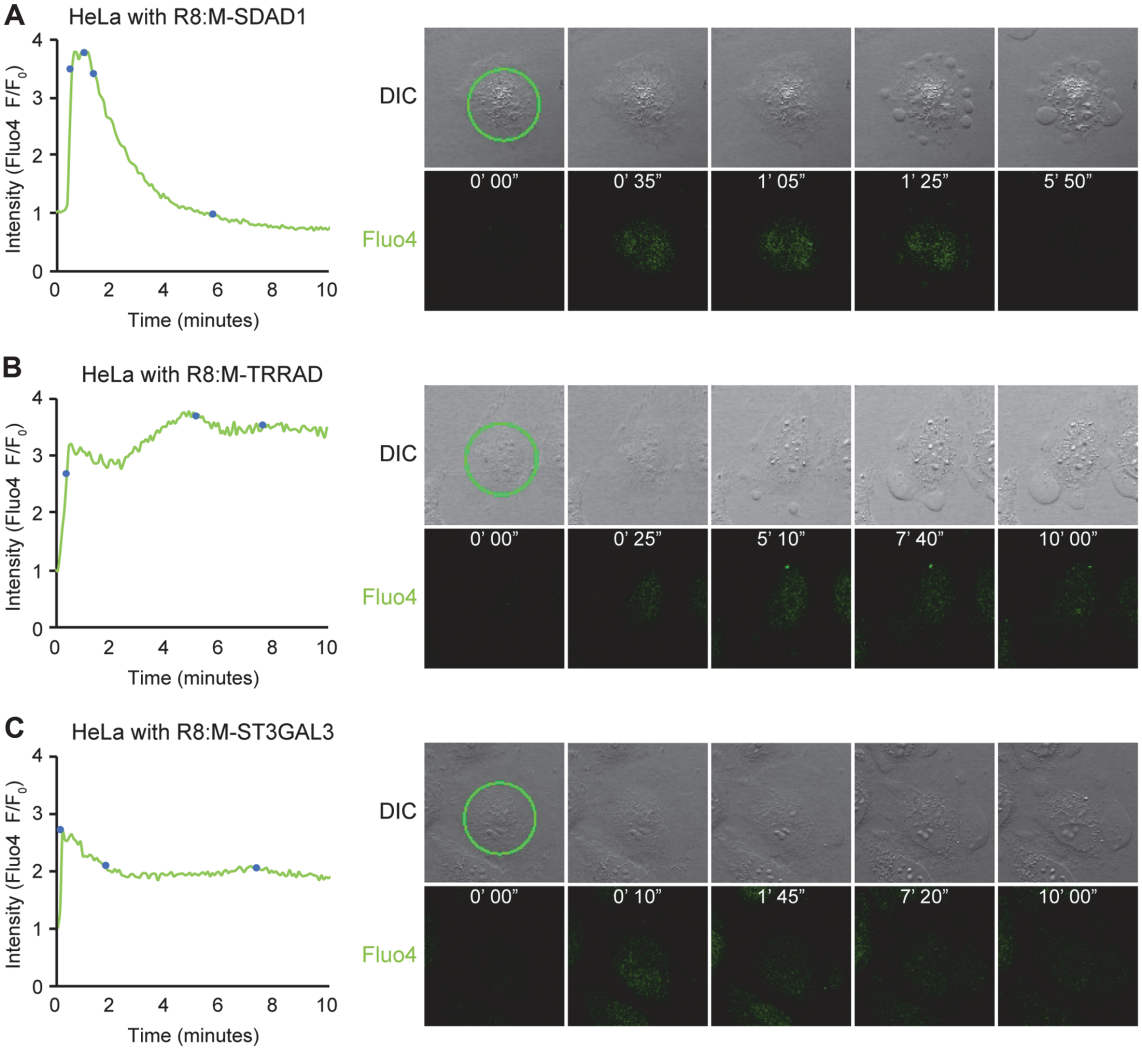
Cytotoxic mitochondrial targeting domain (MTD) homologs induce intracellular calcium influx. HeLa cells were incubated with calcium indicator Fluo-4-AM (5 μM) for 10 min before treatment with cytotoxic mitochondrial targeting domain homologs fused with octa-arginine (R8) to visualize intracellular calcium concentration. After treatment with R8:M-SDAD1 (5 μM, **A**), R8:M-TRRAD (20 μM, **B**), and R8:M-ST3GAL3 (40 μM, **C**), the intensity of Fluo-4 was measured using confocal microscopy (one picture every 5 s) with 488 nm argon laser. Cell morphology was examined using differential interference contrast microscopy.

To compare calcium influx patterns, we introduced calcium indicator Fluo-4-AM and cytotoxic peptides (R8:M-TRRAD, R8:M-ST3GAL3, and R8:M-SDAD1) to HeLa cells and measured the intensity of Fluo-4 using confocal microscopy with argon laser. Treatment with R8:SDAD1 to HeLa cells showed a pattern most similar to that of R8:MTD (Fig. 2A). Immediately after the treatment, the intensity of Fluo-4 sharply increased but no clear morphological changes were observed. At the plateau, some blebs began to form; as the blebs grew larger, the intensity of Fluo-4 decreased slowly. Treatment with R8:M-TRRAD (Fig. 2B) and R8:M-ST3GAL3 (Fig. 2C) into HeLa cells showed quite different patterns. Just after the treatment, similar to the changes observed with the treatment of R8:SDAD1, the fluorescence intensity increased rapidly without morphological changes. However, despite the progression of bleb formation, the fluorescence intensity was maintained. Considering the previous studies, the bleb formation could be due to the initiation of cell membrane disruption, while the dissolution of Fluo-4 could indicate leakage of cytosol contents [7]. Taken together, these cytotoxic peptides may damage the cell membrane enough to penetrate into the cytosol, cause blebs, and induce intracellular calcium influx and cell death. However, R8:M-TRRAD and R8:M-ST3GAL3 may not be strong enough to induce the leakage of cytosol contents, contrary to R8:M-SDAD1. If the mechanism by which these peptides cause calcium inflow is the same as that of R8:MTD, calcium may originate from the mitochondria, ER, and extracellular space.

### Cytotoxic MTD Homologs Induce Mitochondrial ROS Generation

**Fig. 3:**
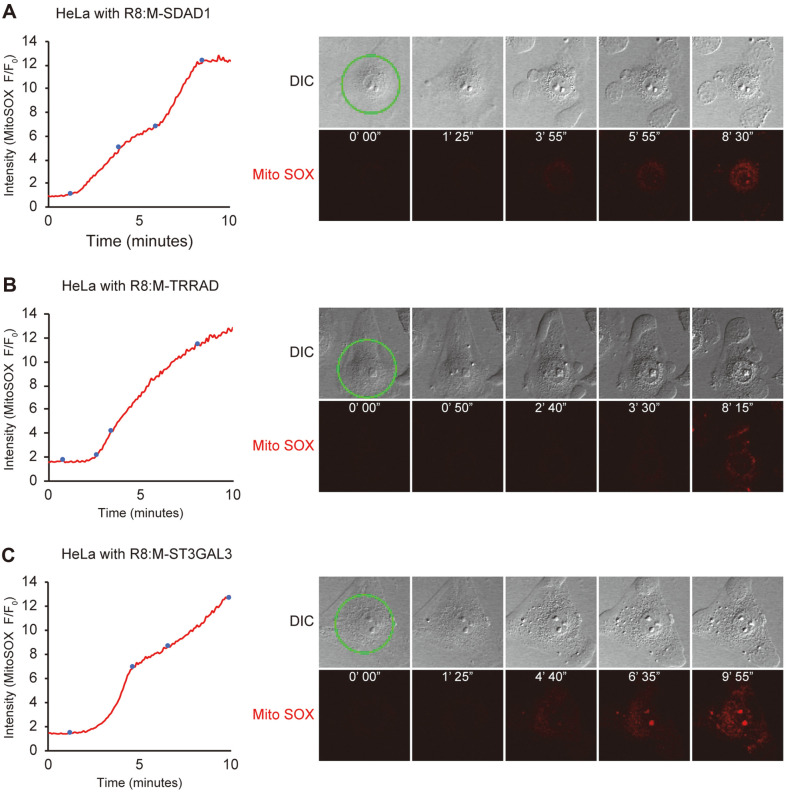
Cytotoxic mitochondrial targeting domain (MTD) homologs induce mitochondrial reactive oxidative species (ROS) generation. HeLa cells were incubated with mitochondrial ROS indicator MitoSOX (5 μM) for 10 min before treatment with cytotoxic mitochondrial targeting domain homologs fused with octa-arginine (R8) to visualize mitochondrial ROS formation. After treatment with R8:M-SDAD1 (5 μM, **A**), R8:M-TRRAD (20 μM, **B**), and R8:M-ST3GAL3 (40 μM, **C**), the intensity of MitoSOX was measured using confocal microscopy (one picture every 5 s) with 488 nm argon laser. Cell morphology was examined using differential interference contrast microscopy.

In addition to MTD-induced cell death, mitochondrial ROS formation plays an important role in other cell death mechanisms, such as apoptosis and necrosis. Therefore, we assumed that these cytotoxic MTD homologs might also induce mitochondrial ROS formation. To measure the mitochondrial ROS level, we treated HeLa cells with the mitochondrial ROS indicator MitoSOX and the cytotoxic MTD homologs, and then measured the fluorescence intensity using time-lapse confocal microscopy. Cytotoxic MTD homologs like R8:M-SDAD1 (Fig. 3A), R8:M-TRRAD (Fig. 3B), and R8:M-ST3GAL3 (Fig. 3C) induced mitochondrial ROS after bleb formation, similar to that in MTD and B1MLM [7, 11]. These results indicate that these peptides may penetrate into the cytosol and induce mitochondrial ROS formation, although it is not certain whether the formation of mitochondrial ROS is a direct cause or result of cell death.

### Cytotoxic MTD Homologs Induce ER Disruption but Not that of Peroxisomes

**Fig. 4:**
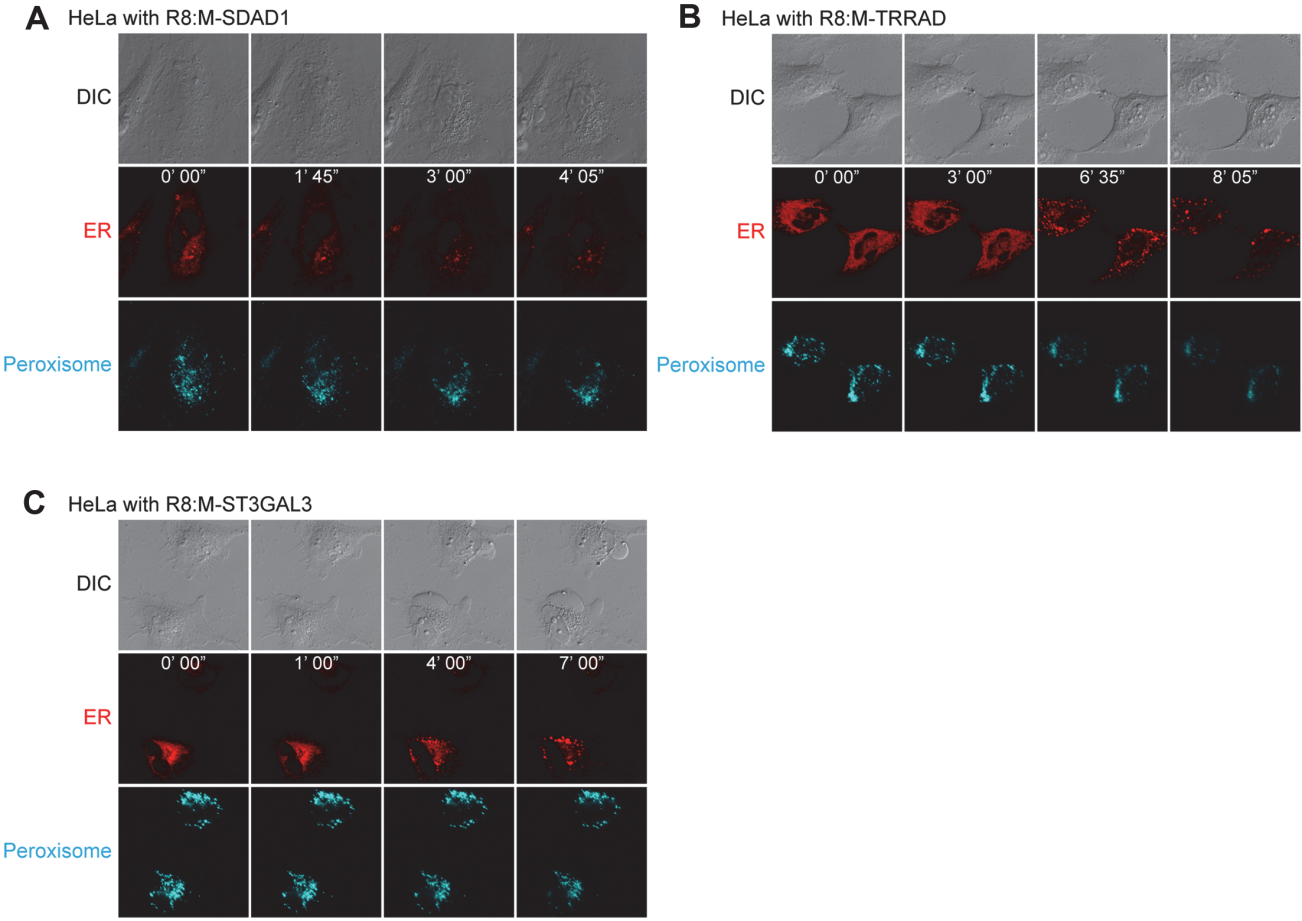
Cytotoxic mitochondrial targeting domain homologs induce endoplasmic reticulum (ER) disruption but not of peroxisomes. To observe ER and peroxisomes, HeLa cells were transfected with pEF.MYC.ER-E2-Crimson and pmTurquoise2-Peroxi. After treatment with R8:M-SDAD1 (5 μM, **A**), R8:M-TRRAD (20 μM, **B**), and R8:M-ST3GAL3 (40 μM, **C**), HeLa cells were observed using confocal and differential interference contrast microscopy with 405 nm and 594 nm laser.

In a previous study, we showed that R8:MTD induced the disruption of ER and mitochondria, as the binding target of MTD is VDAC, and VDAC is also expressed on the ER and mitochondria membranes [7]. Therefore, we assumed that these cytotoxic MTD homologs might induce disruption not of peroxisomes but of ER. To visualize both ER and peroxisomes, we transfected HeLa cells with pEF.MYC.ER-E2-Crimson and pmTurquoise2-Peroxi and observed the cells using confocal microscopy. The MTD homologs like R8:M-SDAD1 (Fig. 4A), R8:M-TRRAD (Fig. 4B), and R8:M-ST3GAL3 (Fig. 4C), as expected, did not disrupt peroxisomes but ER, after the appearance of membrane blebs. These results demonstrated that the cytotoxic MTD homologs disrupt mitochondria and ER, thus implying that their target could be VDACs as well.

## Conclusion

The many homologs of Noxa protein MTD show different cytotoxicity. In addition to previously reported leucine (fifth and ninth) residues, phenylalanine (tenth) seems to be critical in maintaining cytotoxicity as well. R8:M-SDAD1, R8:M-TRRAD, and R8:M-ST3GAL3 showed strong cytotoxicity, with a mechanism, which may be the same as that adopted by R8:MTD. The intracellular calcium spike, mitochondrial ROS formation, and ER disruption induced by R8:M-SDAD1, R8:M-TRRAD, and R8:M-ST3GAL3 in HeLa cells were inhibited by DIDS. These results imply that these homologs could bind to VDACs as well. Amino acid replacement studies to investigate the peptide sequences of the homologs may be helpful to synthesize cytotoxic peptides and artificially control their cytotoxicity for medical applications, e.g., medicines for cancer or other diseases. Furthermore, the possibility of MTD homologs binding to mitochondria and ER could be a new path to reveal other protein functions, such as induction of cell death.

## Figures and Tables

**Table 1:** Peptide sequences.

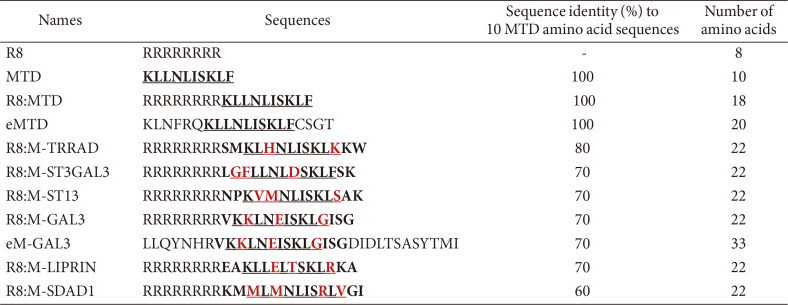

The motifs similar or identical to MTD in peptides were underlined and unmatched amino acid residues in peptides to MTD sequences were indicated as red characters.

## References

[1] Oda E, Ohki R, Murasawa H, Nemoto J, Shibue T, Yamashita T (2000). Noxa, a BH3-only member of the Bcl-2 family and candidate mediator of p53-induced apoptosis. Science (New York, NY.).

[2] Seo YW, Shin JN, Ko KH, Cha JH, Park JY, Lee BR (2003). The molecular mechanism of Noxa-induced mitochondrial dysfunction in p53-mediated cell death. J. Biol. Chem..

[3] Guikema JE, Amiot M, Eldering E (2017). Exploiting the pro-apoptotic function of NOXA as a therapeutic modality in cancer. Expert. Opin. Ther. Targets.

[4] Cheng EH, Wei MC, Weiler S, Flavell RA, Mak TW, Lindsten T (2001). BCL-2, BCL-X(L) sequester BH3 domain-only molecules preventing BAX- and BAK-mediated mitochondrial apoptosis. Mol. Cell.

[5] Tait SW, Green DR (2013). Mitochondrial regulation of cell death. Cold Spring Harb. Perspect. Biol..

[6] Han JH, Park J, Myung SH, Lee SH, Kim HY, Kim KS (2019). Noxa mitochondrial targeting domain induces necrosis via VDAC2 and mitochondrial catastrophe. Cell Death Dis..

[7] Park J, Han JH, Myung SH, Kang H, Cho JY, Kim TH (2019). A peptide containing Noxa mitochondrial-targeting domain induces cell death via mitochondrial and endoplasmic reticulum disruption. Biochem. Biophy. Res. Commun..

[8] Park J, Han JH, Myung SH, Kim TH (2020). Isothiocyanate groups of 4,4'-diisothiocyanatostilbene-2,2'-disulfonate (DIDS) inhibit cell penetration of octa-arginine (R8)-fused peptides. J. Pept. Sci..

[9] Seo YW, Woo HN, Piya S, Moon AR, Oh JW, Yun CW (2009). The cell death-inducing activity of the peptide containing Noxa mitochondrial-targeting domain is associated with calcium release. Cancer Res..

[10] Woo HN, Seo YW, Moon AR, Jeong SY, Jeong SY, Choi EK (2009). Effects of the BH3-only protein human Noxa on mitochondrial dynamics. FEBS Lett..

[11] Park J, Han JH, Myung SH, Seo YW, Kim TH (2018). MTD-like motif of a BH3-only protein, BNIP1, induces necrosis accompanied by an intracellular calcium spike. Biochem. Biophys. Res. Commun..

[12] Boyd JM, Malstrom S, Subramanian T, Venkatesh LK, Schaeper U, Elangovan B (1994). Adenovirus E1B 19 kDa and Bcl-2 proteins interact with a common set of cellular proteins. Cell.

[13] Zhang H, Heim J, Meyhack B (1999). Novel BNIP1 variants and their interaction with BCL2 family members. FEBS Lett..

[14] Ryu SW, Choi K, Yoon J, Kim S, Choi C (2012). Endoplasmic reticulum-specific BH3-only protein BNIP1 induces mitochondrial fragmentation in a Bcl-2- and Drp1-dependent manner. J. Cell. Physiol..

[15] Melikov K, Hara A, Yamoah K, Zaitseva E, Zaitsev E, Chernomordik LV (2015). Efficient entry of cell-penetrating peptide nonaarginine into adherent cells involves a transient increase in intracellular calcium. Biochem. J..

